# Supplementary material for: Cholesteric Liquid Crystals with Thermally Stable Reflection Color from Mixtures of Completely Etherified Ethyl Cellulose Derivative and Methacrylic Acid
Source: Polymers (Basel). 2024 Jan 31;16(3):401. doi: 10.3390/polym16030401 (PMC10856897; doi:10.3390/polym16030401)
Supplement: Supplementary file 1 [file polymers-16-00401-s001.zip › 20240131_polymers-2842487_Suppl Mater_Matsumoto.pdf]

Supplementary Materials

Cholesteric Liquid Crystals with Thermally Stable Reflection Color from Mixtures of Completely Etherified Ethyl Cellulose Derivative and Methacrylic Acid

*Kazuma Matsumoto, Naoto Iwata and Seiichi Furumi**

Department of Chemistry, Graduate School of Science,

Tokyo University of Science, 1-3 Kagurazaka, Shinjuku, Tokyo 162-8601, Japan

*furumi@rs.tus.ac.jp

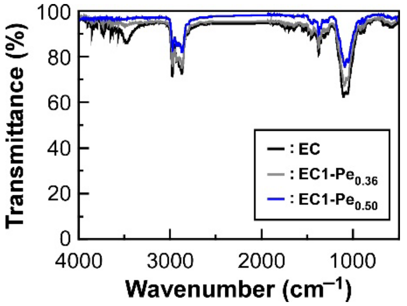

Figure S1. FT-IR spectra of pristine EC (black line), EC1-Pe$_{0.36}$ (gray line), and EC1-Pe$_{0.50}$ (blue line).

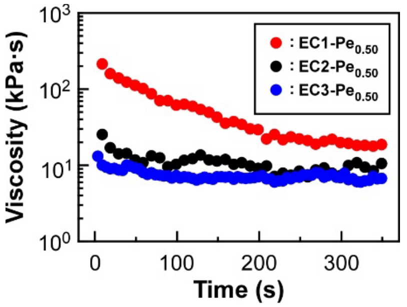

Figure S2. Changes in the viscosity of EC1-Pe$_{0.50}$ (red points), EC2-Pe$_{0.50}$ (black points), and EC3-Pe$_{0.50}$ (blue points) as a function of shearing time.

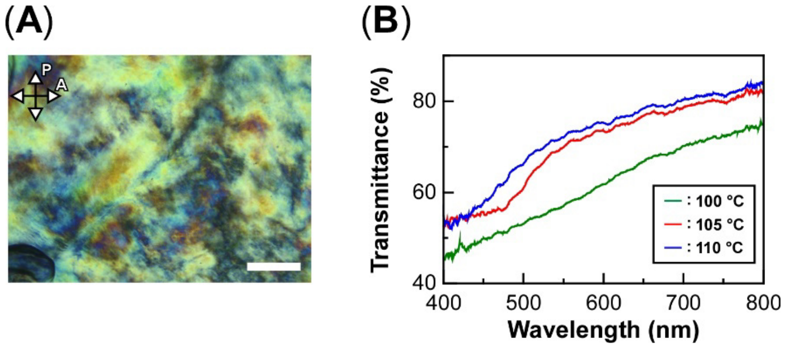

Figure S3. (**A**) Polarized optical microscopic image at 30 °C of **Sample 16** under cross-Nicols. White scale bar denotes 500 μm. The arrows in the upper left corner indicate the directions of polarizer and analyzer. (**B**) Transmission spectra of **Sample 16** measured at 100 °C (green line), 105 °C (red line), and 110 °C (blue line).

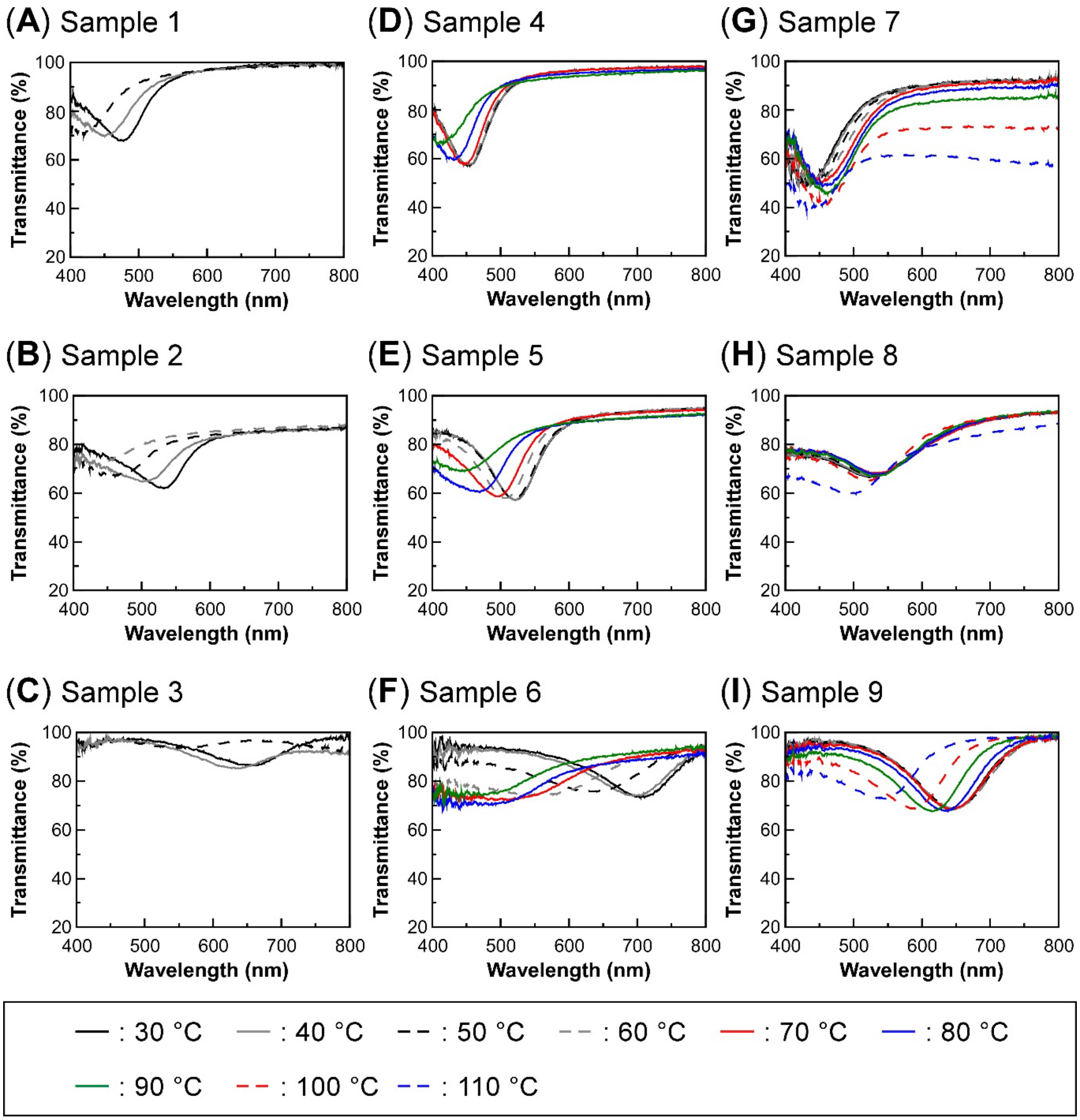

Figure S4. Changes in transmission spectra of these lyotropic CLCs upon heating process. (**A**) **Sample 1**. (**B**) **Sample 2**. (**C**) **Sample 3**. (**D**) **Sample 4**. (**E**) **Sample 5**. (**F**) **Sample 6**. (**G**) **Sample 7**. (**H**) **Sample 8**. (**I**) **Sample 9**.

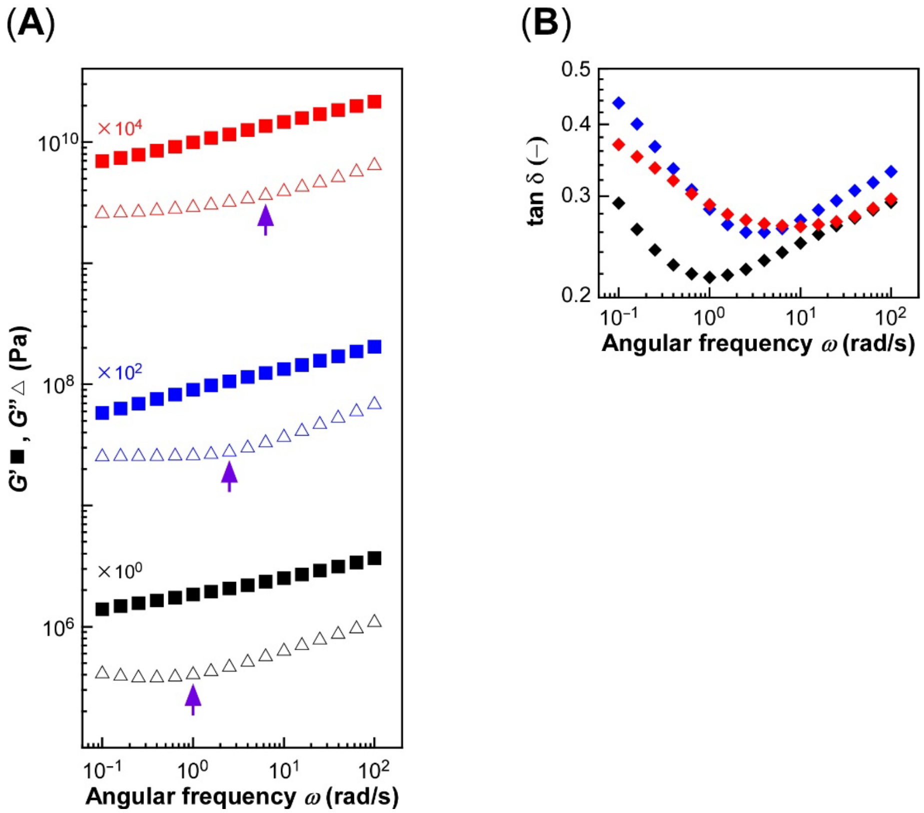

Figure S5. (**A**) Angular frequency (ω) dependence of the storage modulus (G'; closed squares) and loss modulus (G"; open triangles) of **Sample 1** (red plots), **Sample 4** (blue plots), and **Sample 7** (black plots) measured at 30°C. Note that the G' and G" of **Samples 1** and **4** are shifted in the vertical direction of profile values for the reader's clarity. The purple arrows are the inflection points of G". (**B**) Angular frequency (ω) dependence of the loss tangent (G"/G') of **Sample 1** (red plots), **Sample 4** (blue plots), and **Sample 7** (black plots) measured at 30°C.
